# Task-Specific and General Cognitive Effects in Chiari Malformation Type I

**DOI:** 10.1371/journal.pone.0094844

**Published:** 2014-04-15

**Authors:** Philip A. Allen, James R. Houston, Joshua W. Pollock, Christopher Buzzelli, Xuan Li, A. Katherine Harrington, Bryn A. Martin, Francis Loth, Mei-Ching Lien, Jahangir Maleki, Mark G. Luciano

**Affiliations:** 1 Department of Psychology, University of Akron, Akron, Ohio, United States of America; 2 Conquer Chiari Research Center, University of Akron, Akron, Ohio, United States of America; 3 Department of Mechanical Engineering, University of Akron, Akron, Ohio, United States of America; 4 School of Psychological Science, Oregon State University, Corvallis, Oregon, United States of America; 5 Neurological Center for Pain, Cleveland Clinic Foundation, Cleveland, Ohio, United States of America; 6 Department of Neurological Surgery, Cleveland Clinic Foundation, Cleveland, Ohio, United States of America; University of Lethbridge, Canada

## Abstract

**Objective:**

Our objective was to use episodic memory and executive function tests to determine whether or not Chiari Malformation Type I (CM) patients experience cognitive dysfunction.

**Background:**

CM is a neurological syndrome in which the cerebellum descends into the cervical spine causing neural compression, severe headaches, neck pain, and number of other physical symptoms. While primarily a disorder of the cervico-medullary junction, both clinicians and researchers have suspected deficits in higher-level cognitive function.

**Design and Methods:**

We tested 24 CM patients who had undergone decompression neurosurgery and 24 age- and education-matched controls on measures of immediate and delayed episodic memory, as well as three measures of executive function.

**Results:**

The CM group showed performance decrements relative to the controls in response inhibition (Stroop interference), working memory computational speed (Ospan), and processing speed (automated digit symbol substitution task), but group differences in recall did not reach statistical significance. After statistical control for depression and anxiety scores, the group effects for working memory and processing speed were eliminated, but not for response inhibition. This response inhibition difference was not due to overall general slowing for the CM group, either, because when controls' data were transformed using the linear function fit to all of the reaction time tasks, the interaction with group remained statistically significant. Furthermore, there was a multivariate group effect for all of the response time measures and immediate and delayed recall after statistical control of depression and anxiety scores.

**Conclusion:**

These results suggest that CM patients with decompression surgery exhibit cognitive dysfunction compared to age- and education-matched controls. While some of these results may be related to anxiety and depression (likely proxies for chronic pain), response inhibition effects, in particular, as well as a general cognitive deficit persisted even after control for anxiety and decompression.

## Introduction

**Figure 1:**
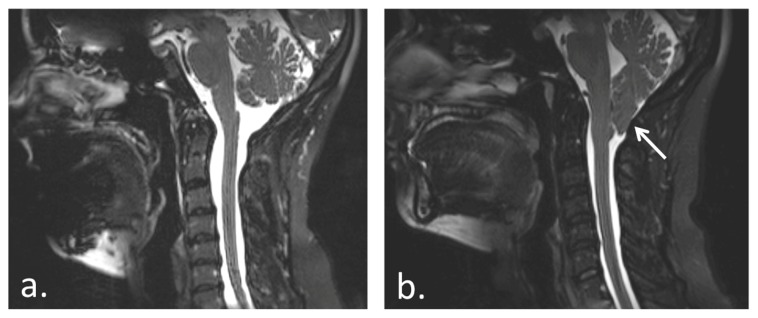
T2-weighted mid-sagittal MRI scan of (a) a healthy subject (b) and Chiari Type I malformation patient with arrow indicating location of tonsillar herniation through the foramen magnum and an asterisk indicating the medullary (brainstem) compression.

Chiari Malformation Type I (CM), affecting approximately 300,000 individuals in the USA, is approximately as common as multiple sclerosis (MS).[1], [2] CM is a clinical syndrome in which the cerebellar tonsils are displaced/descend by 5 mm or greater caudal to the foramen magnum[3], [4] (Figure 1). Even though neuroimaging technologies have led to the improvement of anatomical diagnoses, little is known about the incidence of cognitive symptoms, if any, associated with this syndrome.

While headache and neck pain are the most common symptoms in CM [5], CM patients also may show motoric and cognitive symptoms [3], [6], although studies using precise tests of these potential cognitive deficits are uncommon. Cognitive deficits in CM may result from direct injury of cerebellar [7]–[12] or brainstem [13] systems, or from less direct effects based on anxiety and depression which are commonly seen in CM patients with chronic pain [14]–[19]. In the present study, anxiety and depression were also measured and used as covariates. Note that this argument does require certain assumptions. For example, general pain as an illness is more prevalent than anxiety and depression taken together, although some, but not all, patients with pain end up developing anxiety/depression. However, if we can show that anxiety and depression are significantly correlated with pain in CM patients, then it seems reasonable to use this as a starting place for separating pain-related and other predictors of potential cognitive deficits in CM. However, if group differences (CM vs. controls) in cognition persist after depression and anxiety are covaried out, then other causes of observed cognitive deficit(s) will need to be considered.

We hypothesize that CM patients will show executive dysfunction and episodic memory deficits relative to age- and education-matched controls. However, because both fiber-tract damage and chronic pain models of cognitive dysfunction involve similar brain areas (the cerebellum and the prefrontal cortex), it is difficult to separate fiber-tract damage in CM from chronic pain effects.

### Evidence for Cognitive Deficits in Chiari Malformation

Our present hypothesis is that the downward herniation of the cerebellar tonsils (and/or their cardiac-cycle-based compression of the medulla) result either in direct pressure-related structural damage to the regional neural circuitry and/or cause dysfunction by generating chronic disorders such as pain. We further hypothesize that such damage to the cerebellum and its afferent/efferent circuits can result in cognitive deficits in executive function and episodic memory [12]–[20]. However, published evidence for cognitive deficits resulting from CM is surprisingly limited even though such deficits are hinted at in Yassari and Frim [6].

Kumar et al. [3] reported a neuroimaging study using diffusion tensor imaging (DTI) and intelligence testing on 10 CM patients and 10 controls. Kumar et al. observed that CM patients exhibited decreased fractional anisotropy (or FA) in the genu, splenium, fornix, and cingulum (areas of the brain that connect the limbic system to the medial temporal lobes). Given this location of decreased white-matter integrity, one might expect a CM-related deficit in episodic memory. Kumar et al. also observed cognitive deficits on the picture connection test, digit symbol, block design, picture arrangement, and 5-object assembly test (Wechsler Adult Intelligence Scale, or WAIS), as well as the Trail-Making B test, but no tests of episodic memory were administered. Kumar et al. also did not assess depression and anxiety, and as noted earlier, these variables are correlated with chronic pain that is a key symptom of CM patients. Thus, the present study was conducted to extend the Kumar et al. study to a new set of cognitive tasks to further test for cognitive dysfunction in CM.

### Issues in the Diagnosis of Chiari Malformation Type I

Additionally, it is not clear whether the Kumar et al. [3] CM patients had undergone decompression surgery or were candidates for such a procedure. Because there have been no previous “comprehensive” tests of cognitive dysfunction in Chiari I Malformation that included measures of episodic memory and response inhibition, we felt that it was important in the present study that we obtain a conclusive diagnosis on CM. This is because diagnosis of CM is still somewhat ambiguous—even though it typically requires a 5 mm decension of the cerebellar tonsils into the cervical spinal canal, many neurologists and neurosurgeons also require observable symptoms, such as headache, and that there also be MRI evidence of CSF blockage and medulary compression.

One of the most direct methods to optimize the likelihood that an individual really does have conclusive CM is to select participants who have undergone posterior cranial fossa decompression surgery. CM can first present during childhood or adulthood, although pediatric-onset CM may show differences from adult-onset CM, both age groups frequently receive the same surgical intervention called craniospinal decompression surgery [21]. This surgery entails bone removal in the posterior cranial fossa, to varying degrees, along with the upper arch of the C1, and sometimes the C2, vertebrae. The objective is to restore space at the craniospinal junction in order to relieve the direct pressure on the brain stem and cerebellum. However, one consequence of this method is that the surgical procedure itself (rather than CM) could potentially result in cognitive dysfunction. However, most neurosurgeons feel that this procedure tends to alleviate symptoms associated with CM (e.g., headache), so it is likely that our present approach is a more conservative test of cognitive dysfunction in CM than using pre-decompression-surgery participants. An added benefit to the present approach is that if we were to use candidates for decompression surgery (who have not yet had surgery), patients' anxiety and/or depression might have been elevated due to the uncertainty of imminent neurosurgery. Thus, we decided that the optimal method for a comprehensive test of cognitive dysfunction in CM should use individuals who have already undergone decompresion surgery (at least six months prior to cognitive testing).

### The Present Study

The present study examined the cognitive performances of CM patients who had undergone decompression surgery in addition to a sample of age- and education-matched, healthy controls. A secondary goal of this study was to use statistical control methods to distinguish between measures of anxiety and depression (likely related to chronic pain), and fiber-tract damage accounts of cognitive symptoms. To assess cognitive performance, we used a variation of the Rey Auditory Verbal Learning Test (RAVLT; a test of immediate and delayed episodic memory) using non-timed written responses and three computerized measures of executive function: an automated digit symbol substitution task [22] (a measure of processing speed with some memory load), a Stroop interference task [23] (a measure of response inhibition), and the Operation Span task (or Ospan, a measure of working memory) [24] using timed, button-press responses from a computer keyboard. To assess anxiety, depression, and stress levels in all participants, we used the 21-item, self-report Depression Anxiety and Stress Scale (DASS21) [25]. To directly assess self-reported head and neck pain in CM patients, we used the self-report Neck Pain Disability Index Questionnaire [26].

## Methods

### Ethics Statement

The present study was approved by the University of Akron Institutional Review Board (Akron, Ohio) and all participants (or their guardians) provided written informed consent.

### Participants

Twenty-four CM patients (22 females, 2 males) who had undergone decompression surgery (age range: 15–59 years, mean age = 38.6 years, mean education = 14.6 years) and 24 age- and education-matched controls (15 females, 9 males; age range: 15–56 years, mean age = 39.2 years, mean education = 15.1 years) participated in the present study. There were no group differences in either age, F(1, 46) = .03, p = .86, or in years of education, F(1, 46) = .50, p = .48.

We selected post-decompression CM patients in order to assess potential cognitive deficits in more severe cases. All of these CM participants had considerable MRI evidence of cerebellar herniation below the foramen magnum in addition to being symptomatic with headache, dizziness and/or balance issues. In order to make sure that postoperative recovery was not contributing to the present results, we required at least a six-month interval between decompression surgery and participation in the present study. Approximately 80% of pre-decompression surgery CM patients experience severe headaches [5]–[6], and many of these patients are given opiate-based analgesics (e.g., Vicodin). However, because such analgesics can have an effect on cognition and/or contribute to ongoing headache in the long run, we limited participation in the present study of post-decompression participants to individuals who used just anti-inflammatories (NSAIDs) and acetaminophen (no opiate-based analgesics). In selecting post-decompression CM patients, though, we understand that we may have underestimated some CM cognitive deficits secondary to recovery.

### Tasks and Procedure

Participants were tested individually on a computer and completed all of the tasks in one session. Each session began with immediate recall, followed by the digit symbol, Stroop, and working memory tasks assessing executive function. Finally, participants then completed the delayed recall task, followed by the depression, anxiety, and stress paper-and-pencil assessments. In addition, the CM patients but not the controls completed a pain and disability survey after the other tasks. The total testing time was approximately one hour.

#### RAVLT

To assess performance on episodic memory recall [27], we used a modified version of the Rey Auditory Verbal Learning Test [28]. Participants were presented orally the 15 words individually (approximately one second per word) and were asked to recall the words immediately after the first presentation of all of the words (the immediate recall) and also to recall the words 40 minutes later (the delayed recall) after the participants had completed the three executive function tasks. Participants wrote down their responses for both the immediate and delayed recall tasks, so the dependent variable was the number of written correct responses.

#### Stroop Test

Performance on the Stroop task has commonly been used as one indicator of frontal-lobe function measuring inhibitory control [29], [30]. Specifically, it is important to note that the Stroop task is a measure of prepotent response inhibition [31]. The present Stroop test [32] involved the presentation of a single color word on a computer monitor (either “RED,” “BLUE,” “GREEN,” or “PURPLE”). Words could be printed either in a color that matched the word (congruent trial) or in a different color than the word (incongruent trial). Participants were asked to identify the word or identify the color in which the word was printed. Responses for the four response alternatives were collected through the use of computer keys (the “1,” “2,” “3,” and “4” keys). Reaction and accuracy served as the dependent variables. There were 20 practice trials and 96 experimental trials (48 “word meaning” trials and 48 “color” trials: 24 congruent and 24 incongruent of each).

#### Ospan Test

Working memory is the cognitive system that allows individuals to temporarily hold information in memory and to manipulate this information [33]. The Ospan test [24] is one of the most widely used measures of working memory capacity that includes both short-term memory maintenance (remembering sequences of letter string from 3–7 letters in length) as well as manipulation of math problems. However, it should be noted that the Ospan task has also been commonly used to measure fluid intelligence, which has been found to be correlated with the Raven's Progressive Matrices and mentally rotated blocks [24]. However, as noted in Unsworth et al. (2005, Figure 2) [24], fluid intelligence and working memory capacity (as measured by the Ospan task) form separate latent factors in structural equation models, implying that working memory capacity forms a separate construct from fluid intelligence. Thus, it is reasonable to assume that the Ospan task is a separate measure of working memory capacity independent of fluid intelligence.

**Figure 2:**
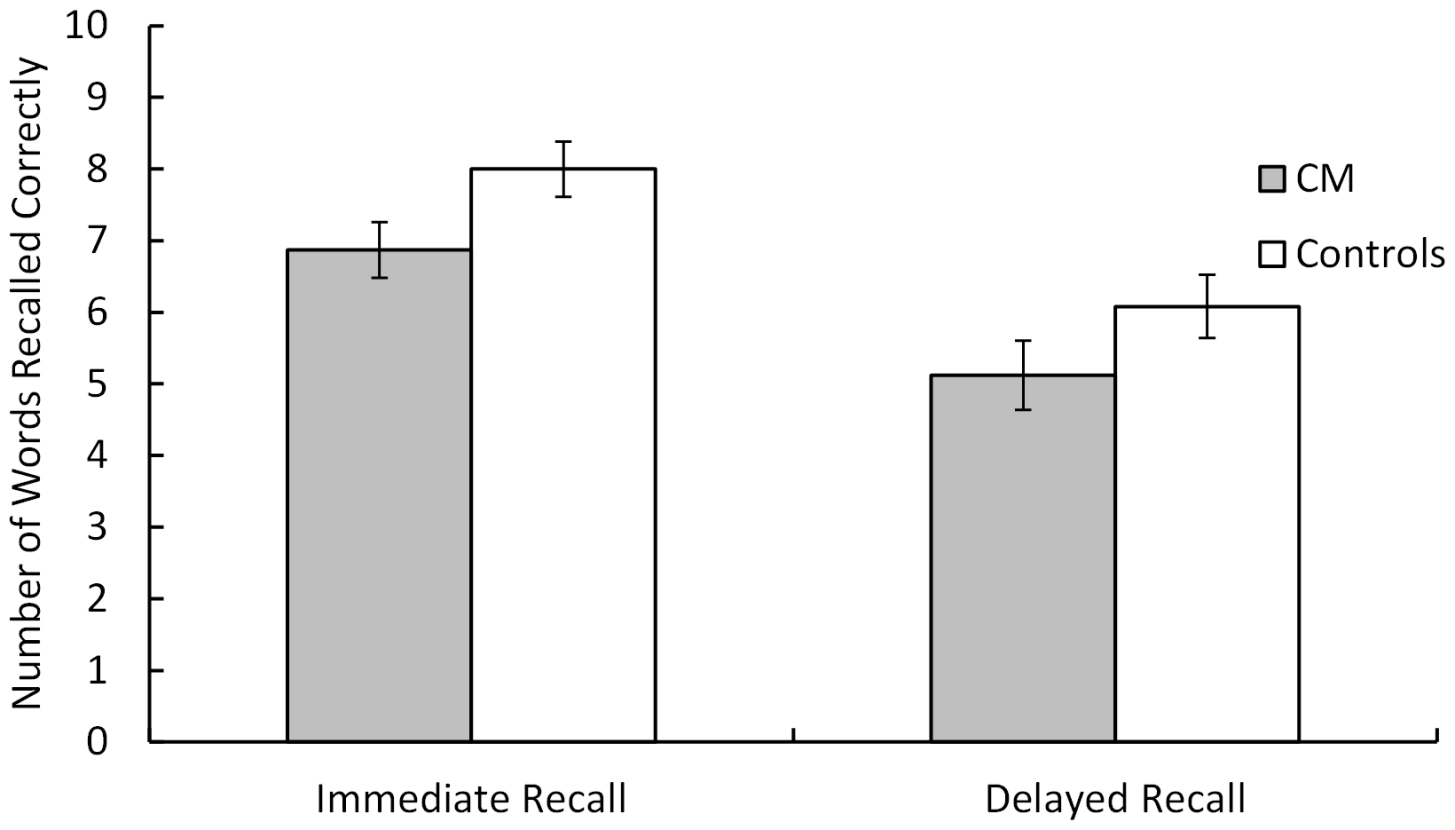
The mean total number of words correctly recalled in the immediate and delayed recall conditions for the Chiari Patients (CM) and Controls. Error bars represent the standard errors of the means.

There were 75 letters and 75 math problems in the presently used automated Ospan task based on Unsworth et al [24]. In the present study we report absolute Ospan scores and a measure of mean RT for the mental arithmetic solution time (the “working” portion of working memory), as well as accuracy of math computations.

#### Digit Symbol Substitution Task

We used a computer-administered version of the digit symbol substitution task [22]. Across the top of the computer screen, nine digits (from 0–9) were presented in a key along with nine symbols. Each digit was associated with a given symbol. In each trial, a single digit-symbol pair was presented directly below the middle of the key. Participants were instructed to respond whether the present pair was correct or incorrect. There were a total of 72 experimental trials.

We used SAS (Version 9.3) and SPSS (IBM SPSS Version 20) software to analyze the present results.

## Results

For the RAVLT analysis, we had a 2 (group: CM vs. controls)×2 (retention interval: immediate vs. delayed recall) mixed design in which group was measured across participants and retention interval was measured within participants. The main effect of group approached significance, F(1, 46) = 3.46, p = .07, η_P_^2^ = .07 (words recalled: CM group = 6.00, Control group = 7.04, and there was a main effect of retention interval, F(1, 46) = 67.26, p<.0001, η_P_^2^ = .60 (immediate recall = 7.44 words, delayed recall = 5.60 words), but group did not interact with retention interval (p = .71) (see Figure 2).

**Figure 3:**
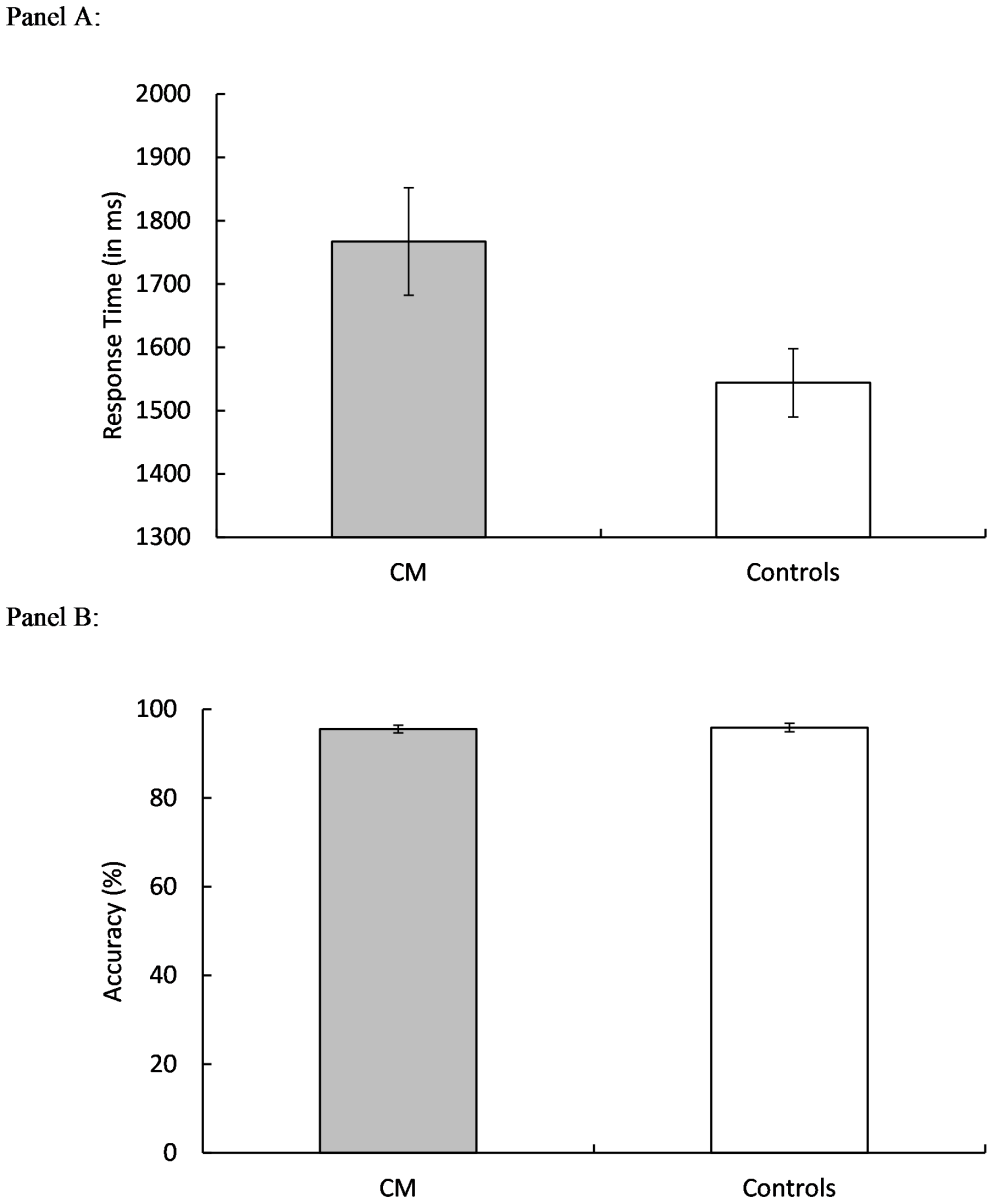
The mean response time (Panel A) and accuracy (Panel B) in digit symbol substitution task for the Chiari Patients (CM) and Controls. Error bars represent the standard errors of the means.

For the digit symbol substitution task [22], a measure of processing speed, we compared means across group for response time (RT, in milliseconds) and accuracy (in mean percent error). There was a main effect of group for RT, F(1, 46) = 4.95, p = .03, η_P_^2^ = .097, (CM = 1767 ms, controls = 1544 ms) (Figure 3), but there was no main effect for accuracy (p = .80).

**Figure 4:**
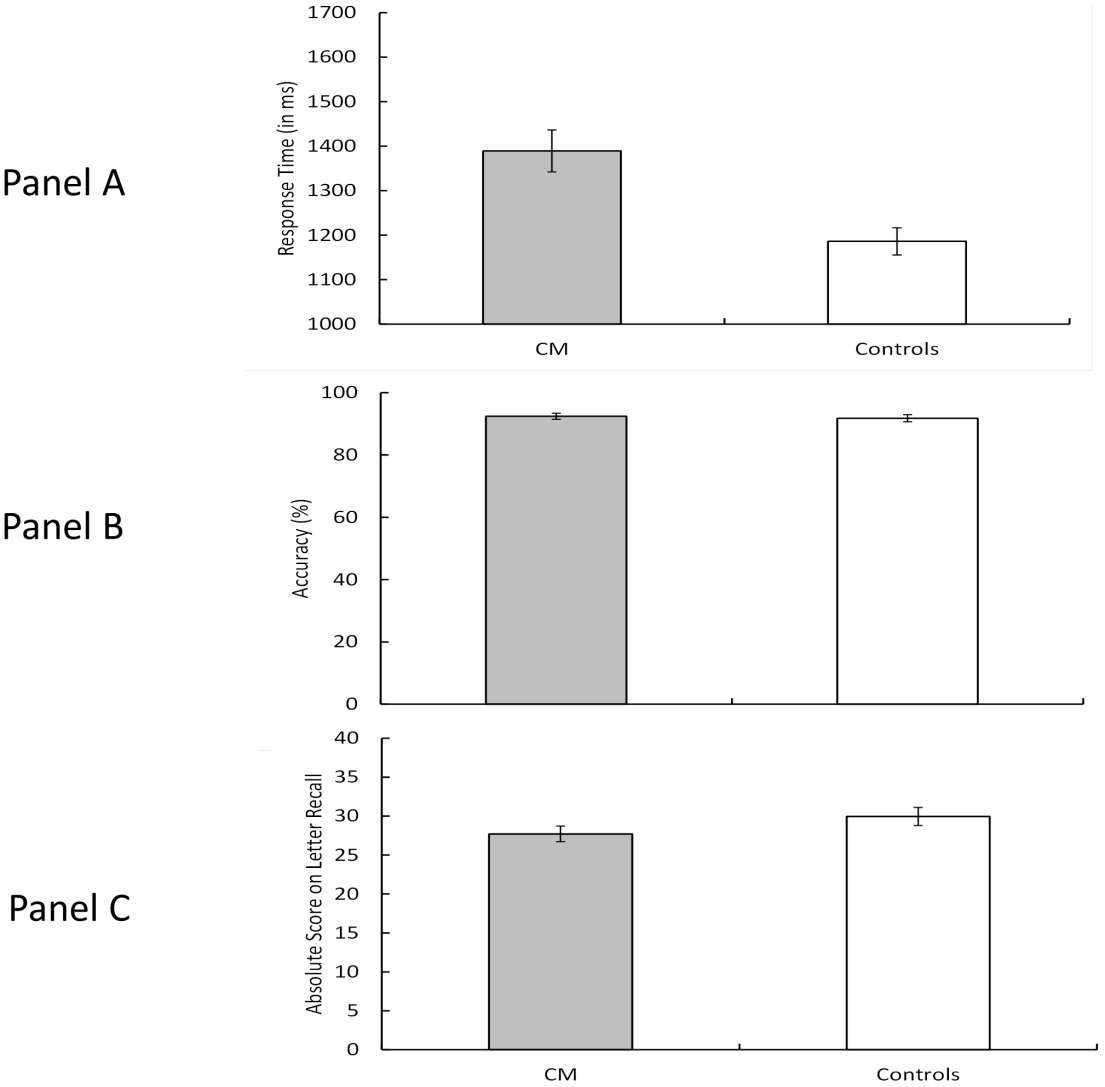
The mean response time (Panel A) and accuracy (Panel B) in Automated Operation Span (Ospan) computation time and accuracy as well as the total number of letters correctly recalled (Ospan Absolute Score; Panel C) for the Chiari Patients (CM) and Controls. Error bars represent the standard errors of the means.

The Ospan task [24] is a set of measures of working memory capacity. There was a main effect of group for math computation RT, F(1, 46) = 13.05, p<.001, η_P_^2^ = .18, indicating that the CM group was significantly slower in computing the answers to math problems than were the controls (CM group = 1389 ms, controls = 1186 ms) (Figure 4). There was no effect of group for computational accuracy (p = .69). Also, there was no group effect for the total number of correctly recalled letter sequences (p = .68).

**Figure 5:**
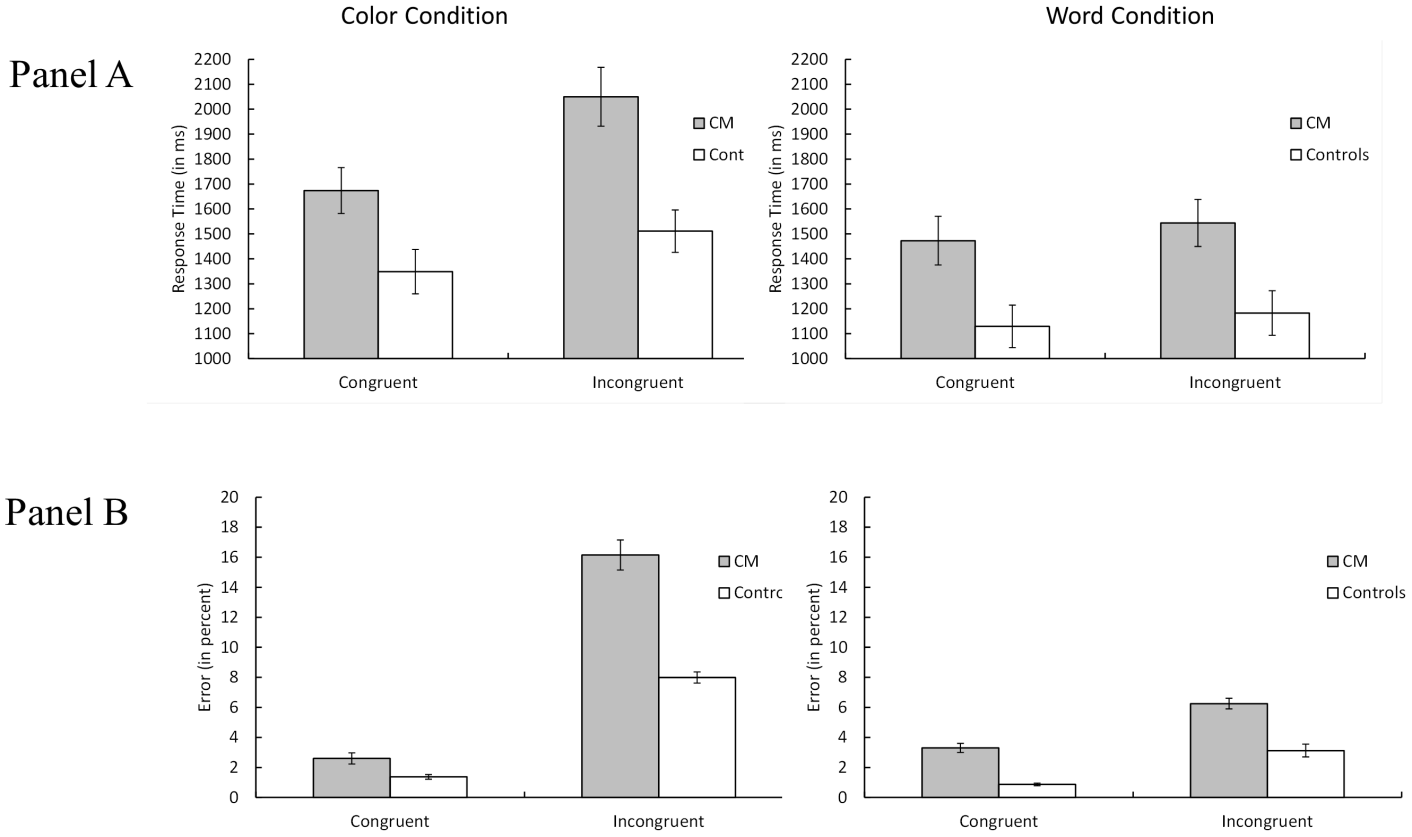
The mean response time (Panel A) and percent error (Panel B) in Stroop task (Color vs. Word) as a function of congruency between color and word (congruent vs. incongruent) for the Chiari Patients (CM) and Controls. Error bars represent the standard errors of the means.

The Stroop task is a measure of response inhibition [23], [31]. A 2 (group; a between-subject variable) ×2 (task type: color vs. word; a within-subject variable) ×2 (congruency: congruent vs. incongruent; a within-subject variable) mixed analysis of variance (ANOVA) was used to analyze the Stroop data. For RT, there were main effects for group, F(1, 46) = 11.58, p<.01, η_P_^2^ = .25 (CM = 1685 ms, controls = 1293 ms), task type, F(1, 46) = 28.21, p<.0001, η_P_^2^ = .06 (color = 1646 ms, word = 1332 ms), and congruency, F(1, 46) = 46.22, p<.0001, η_P_^2^ = .50 (congruent = 1406 ms, incongruent = 1572 ms). The key finding was a Group x Task Type x Congruency interaction, F(1, 46) = 5.65, p<.05, η_P_^2^ = .11, that occurred because the CM group showed a relatively larger congruency effect for the color condition (376 ms) versus the word condition (71 ms), relative to the control group for the color condition (162 ms) versus the word condition (54 ms) (Figure 5). To confirm this interpretation, we ran separate analyses for the Group x Congruency simple effects by task type. The Group x Congruency interaction was significant for color type, F(1, 46) = 12.17, p = .001; but this interaction was not significant for word type, F<1.0.

No effects in the accuracy analysis for the Stroop data reached significance (all p's>.05).

### Generalized Slowing Analyses for the Stroop Task

The observed slower responses for the CM group compared to the control group could be due to generalized slowing, such as psychomotor speed, rather than to task-specific slowing [34]. Madden, Pierce and Allen (1992) [35] reported a method that can be used to examine this possibility. First, one needs to find the best-fitting linear equation for RT_CM_ = mRT_controls_+b (reaction time, or RT, for CM patients should be a linear combination of controls' RT). Because we collected RT data from three different tasks (Stroop, Ospan, and Digit Symbol), in order to consider true general slowing rather than task-specific slowing, we needed to compute the linear slowing function for all three tasks. For the present tasks, this best fitting linear slowing function was RT_CM_ = (1.16)RT_controls_+120 ms, R^2^ = .76. The next step was to transform the controls' RT data from the Stroop task using this linear function. This procedure will eliminate the main effect for group [35], and if task-related slowing is generalized, then the Group x Color Type x Task Type interaction for the Stroop task will also be eliminated [35]. However, if the task-specific slowing for the Stroop task goes beyond that predicted by general slowing, then this three-way interaction should remain statistically significant even after the controls' data are transformed into “generalized” replicas of CM patients' data [35]. When we transformed the controls' RT data for the Stroop task using the aforementioned generalized slowing equation and then added the non-transformed CM patients' data, the main effect for group was no longer significant, F(1, 46) = .28. p = .60. However, the Group x Color Type x Task Type interaction remained statistically significant, F(1, 46) = 4.31, p = .0435. Using the same logic as Madden et al. [35], we can conclude that the present Stroop response inhibition results for Chiari patients relative to controls cannot be accounted for by generalized slowing. Instead, it appears that these results are primarily due to task-specific slowing.

### Depression, Anxiety, and Pain Analyses

Chiari patients, even after decompression surgery, still frequently experience severe headaches. To assess neck pain disability (including headache), we tested just the CM group on the Neck Pain Disability Index Questionnaire [26] (because individuals in the control group would typically score zero). Using the scoring criteria proposed by Fairbanks et al. [26], the present Chiari sample had a percent disability score of 47% (substantial disability due to neck and head pain). Next, we correlated the CM group's pain score with the DASS21 [25] scores (see Table 1 for correlation matrix). Pain and depression (r = .51, p = .01, r^2^ = .26), as well as pain and anxiety (r = .56, p = .006, r^2^ = .31) were significantly correlated, but pain and stress were not (r = .32, p = .12, r^2^ = .10). This indicates that pain, depression, and anxiety (but not stress) scores were significantly related in CM patients.

**Table 1:** Correlation Matrix (Pearson's r) for Just Chiari Malformation Patients (N = 24) for Depression, Anxiety, Stress, Pain, Digit Symbol RT, Ospan RT, the Stroop Congruity Effect for Color, and Immediate Recall.

	Depression	Anxiety	Stress	Pain	DSRT	OspanRT	StroopRT
Depression							
Anxiety	.67*						
Stress	.52*	.49*					
Pain	.51*	.56*	.32				
DSRT	.66*	.52*	.04	.56*			
OspanRT	.46*	.48*	.08	.46*	.66*		
StroopRT	.10	.11	.08	.34	.58*	.55*	
Immediate Recall	−.09	−.01	−.17	−.37	.006	.201	.04

* *p*<.05

The next step was to correlate depression, anxiety, and pain scores for CM patients with immediate recall, digit symbol RT (DSRT), Ospan computation RT, and Stroop congruency effects for the color condition (i.e., the four cognitive variables that showed statistically significant group differences). The correlation matrix for these analyses is presented in Table 1. Depression, anxiety, and pain all showed significant correlations with DSRT and Ospan computational RT, but not with Stroop congruency effects for the color condition or immediate recall. These results suggest that pain scores, depression, and anxiety in the CM group were significantly related to DSRT and working memory computational RT performance, but not with response inhibition (Stroop) or immediate recall performance.

We also compared depression, anxiety, and stress levels across groups (i.e., all 48 participants—not just the 24 CM patients' data) using the DASS21 scale data. The CM group showed significantly higher scores in depression (CM group = 6.5, controls = 2.6), F(1, 46) = 8.48, p<01, η_P_^2^ = .16, anxiety (CM group = 8.6, controls = 1.8), F(1, 46) = 31.79, p<.0001, η_P_^2^ = .41, and stress (CM group = 9.2, controls = 5.3), F(1, 46) = 8.35, p<.01, η_P_^2^ = .15. The present results showing that CM patients show symptoms of depression and anxiety are consistent with the findings of Mueller and Oro [36] who screened a much larger sample of CM patients for symptoms and observed that CM patients showed increases in depression and anxiety.

Because neck and head pain were correlated with cognitive performance in CM patients, and with depression (r = .51) and anxiety (r = .56), we conducted an analysis of covariance (ANCOVA) on the four measures showing significant effects for groups (or interactions with group) in the earlier ANOVAs (see Table 2). For the digit symbol substitution task, F(1, 44) = .086, p = .77, η_P_^2^ = .002, the Ospan RT task, F(1, 44) = 2.46, p = .12, η_P_^2^ = .05, and the immediate recall task, F(1, 44) = 1.83, p = .18, η_P_^2^ = .087, the main effects of group were no longer significant when depression and anxiety were entered as covariates. However, the Group x Task Type x Congruency interaction for the Stroop analysis remained significant, F(1, 44) = 6.69, p<.02, η_P_^2^ = 13, even after depression and anxiety were entered as covariates. These results suggest that variables correlated with chronic pain (i.e., anxiety and depression) accounted for all cognitive deficits in CM except for response inhibition (Stroop) effects.

**Table 2:** Correlation Matrix (Pearson's r) for Chiari Malformation Patients and Controls (N = 48) for Depression, Anxiety, Stress, Digit Symbol RT, Ospan RT, the Stroop Congruity Effect for Color, and Immediate Recall.

	Depression	Anxiety	Stress	Processing Speed	Working Memory	Inhibitory Control
Depression						
Anxiety	.73*					
Stress	.62*	.57*				
Processing Speed	.56*	.49*	.23			
Working Memory	.39*	.50*	.17	.56*		
Stroop RT	.21	.34*	.17	.43*	.47*	
Immediate Recall	−.17	−.22	−.03	−.08	−.24	−.02

*p<.05.

### Multivariate Analyses

Because we have reported results from four different tasks (Stroop, Ospan, Digit Symbol, and episodic memory: immediate and delayed recall), an important issue to consider is whether Chiari patients showed an “overall” cognitive deficit relative to age- and education-matched controls. One way to test for this possibility is to use latency scores from the Stroop, Ospan (math computational speed), and Digit Symbol tasks, and recall data from the memory tasks as dependent variables, and to use group as the independent variable and conduct a multivariate analysis of variance, or MANOVA. When we conducted this MANOVA, the multivariate effect of group was significant, Wilks' Lambda = .58, p = .004. In the univariate “step-down” analyses, all of the dependent variables were statistically significant except for delayed recall. These results indicate that the composite cognitive dependent variable in the present study varied across group. That is, Chiari patients performed significantly more poorly than controls did on global cognitive function. However, in the present study, we also need to consider the effects of anxiety and depression. In particular, was there a multivariate effect of group even after the effects of anxiety and depression are covaried out? The answer to this question is “yes.” Namely, the multivariate analysis of covariance, or MANCOVA showed a statistically significant multivariate effect of group even when anxiety and depression were entered as covariates, Wilks' Lambda = .654, p = .031.

## Discussion

We assessed cognitive performance in CM patients with a firm diagnosis of CM who had undergone decompression surgery (minimum six months prior to testing in the present study). Little is known about the cognitive consequences of CM except for one study by Kumar et al. [3]. In an attempt to gain a more thorough understanding of the cognitive consequences of CM, we measured group differences between CM patients and age- and education-matched controls in response inhibition (Stroop), working memory (Ospan computational speed), processing speed (Digit/Symbol task), and episodic memory performance (modified RAVLT). The present results provide evidence that CM patients showed deficits in response inhibition, working memory speed, and processing speed relative to age- and education-matched controls. Also, CM patients showed deficits in episodic recall that approached statistical significance.

### Cognitive Deficits in CM

With regard to working memory, CM patients did show significantly slower computational responses (Ospan RT) than controls, but group differences for this variable were eliminated when we statistically controlled for depression and anxiety scores as covariates—suggesting that group differences in working memory speed may be accounted for by chronic pain. Processing speed showed similar results—CM patients showed significantly slower digit symbol RT (DSRT) than did controls, but, again, group differences in processing speed were eliminated when we statistically controlled for anxiety and depression effects. On the other hand, response inhibition deficits (as measured by Stroop interference effects) in the CM group persisted even after statistical control of anxiety and depression effects. Furthermore, this color congruency effect was not significantly correlated with pain, depression, or anxiety in the CM group (see Table 1). This appears to be a response inhibition deficit [31].

Response inhibition, a type of attentional guidance [37], is related to selective attention. Human observers focus on information relevant to a task (in the Stroop task, “which response do I select?”), but must filter out (inhibit) non-relevant response information. Thus, a strong emphasis is placed on inhibitory control so that individuals can operate efficiently within this limited-capacity attentional system. Inhibitory control processing is typically associated with the dorsolateral prefrontal cortex and the anterior cingulate cortex [23] as well as areas of the parietal cortex—the frontoparietal attentional pathway [38]. However, it is known that CM is most commonly associated with damage to the cerebellum and brainstem [5], so it seems to suggest that performance deficits associated with the prefrontal cortex would be present. Could it be, then, that the response inhibition component of the Stroop task [31] is actually related to the cerebellar and/or brainstem damage in CM? We cannot conclusively answer this question in the present study because it did not include neuroimaging analyses (e.g., DTI-based tractography or fMRI-based functional connectivity) that would allow an examination of the integrity of fiber tracts connecting the cerebellum and/or brainstem to the front-parietal attentional pathway. However, Hesselmann, Flandin, and Dehaene (2011) [38] did report an fMRI/Event-Related Potential (ERP) study on a task known to have a response selection locus—the psychological refractory period (or PRP) paradigm. When they subtracted single-task from dual-task performance, they found significant activation for just the dual-task (PRP) component in the left middle and superior frontal gyrus areas—essentially Brodmann's area 46—part of the dorsolateral prefrontal cortex. Furthermore, when the fMRI subtracted data (i.e., the task component known to be related to response selection—of which a critical component is response inhibition) were synchronized with the ERP (P3) data, Broadmann's area 46 and areas in the parietal cortex were activated. These results showing that a task known to have a response selection/inhibition locus (the PRP effect) activated the fronto-parietal (or dorsal) attentional pathway suggest that response inhibition shares the same attentional pathway known to affect stimulus selection. This provides inductive evidence that the present response inhibition (Stroop) deficit observed in individuals diagnosed with CM might be associated with a prefrontal cortex deficit, although additional neuroimaging support for this CM assertion is needed to confirm the present hypothesis because there is evidence that the relationship between individual neuropsychological test data and specific brain regions is not necessarily specific [39].

What is not clear from the present study, though, is why CM patients showed specific deficits in response inhibition (Stroop interference), even when the effects of anxiety and depression were statistically controlled for, but not in working memory or processing speed—two other measures of executive function. Perhaps the most parsimonious interpretation is that response inhibition is more closely related to motoric processing known to be associated with cerebellar function (although response inhibition is an attentional process rather than a motoric process, per se) or reflexive processing known to be associated with medullary function. On the other hand, working memory and processing speed do not appear to be as closely associated with cerebellar and/or brainstem function as is response inhibition [8]. A more direct test of this issue would be to assess CM patients on both response inhibition and distractor interference tasks (e.g., an Eriksen flanker task) [31]. If CM patients showed performance decrements on both tasks, then this would provide evidence of a more general executive function deficit. On the other hand, if CM patients showed a deficit on the response inhibition task, but not on the distractor interference task, then this would provide evidence of a more specific deficit perhaps more localized at the cerebellar and/or brainstem level. Thus, while there is good reason to believe that response selection/inhibition, at least as measured by the psychological refractory period effect, shows a clear prefrontal attentional effect [38], it could be that response inhibition is also closely linked to cerebellar and/or medullary processing.

Another issue germane to the seemingly larger Stroop effects for CM patients than for controls is whether this effect was the result of generalized slowing in CM patients. To test for this possibility, we transformed the controls' data using the slowing function taken from the CM patients' data [34], which were then analyzed with the untransformed data from the CM patients. In this analysis, we still observed the Groups x Color Type x Task Type interaction. According to Madden et al. [35], these results suggest that the group-related differences are specific to a given task—not the result of generalized slowing across all tasks (in this case, Stroop, Ospan working memory, and automated digit/symbol). Thus, the presently observed larger response inhibition effects for CM patients relative to controls are the result of task-specific effects.

### Episodic Memory Effects in CM?

Episodic memory is defined as contextual memory (events associated with time-, space-, or emotion-based contexts [27], [40]. We observed marginally poorer episodic recall in individuals diagnosed with CM than in controls in a modified version of the RAVLT [28]. However, the group effect for recall was eliminated after statistical control for anxiety and depression effects. This suggests that the marginally significant group effects in recall were associated with a variable related to anxiety and depression—likely chronic pain.

### Multivariate Effects

Given that all of the cognitive deficits other than the Stroop (response inhibition) effect were eliminated, an important issue to address is whether there was an “overall” cognitive deficit—especially after the effects of anxiety and depression (thought to index chronic pain effects in CM) were controlled. We addressed this issue using MANOVA and MANCOVA analyses. When all of the reaction time and memory recall data were included as dependent variables, and group (CM vs. controls) was included as an independent variable, the resulting MANOVA showed that the main effect of group was significant. However, this multivariate effect for group (i.e., that CM patients showed an overall cognitive performance deficit relative to age- and education-matched controls) could have been the result of anxiety and depression effects. To test for this possibility, we also conducted a multivariate analysis on group while including anxiety and depression as covariates. The resulting MANCOVA showed that the main effect of group remained statistically significant even after we controlled for anxiety and depression. Consequently, an overall cognitive deficit in CM patients was observed that cannot be explained by increased anxiety and depression levels in CM patients.

### Locus of the Cognitive Effects

**Figure 6:**
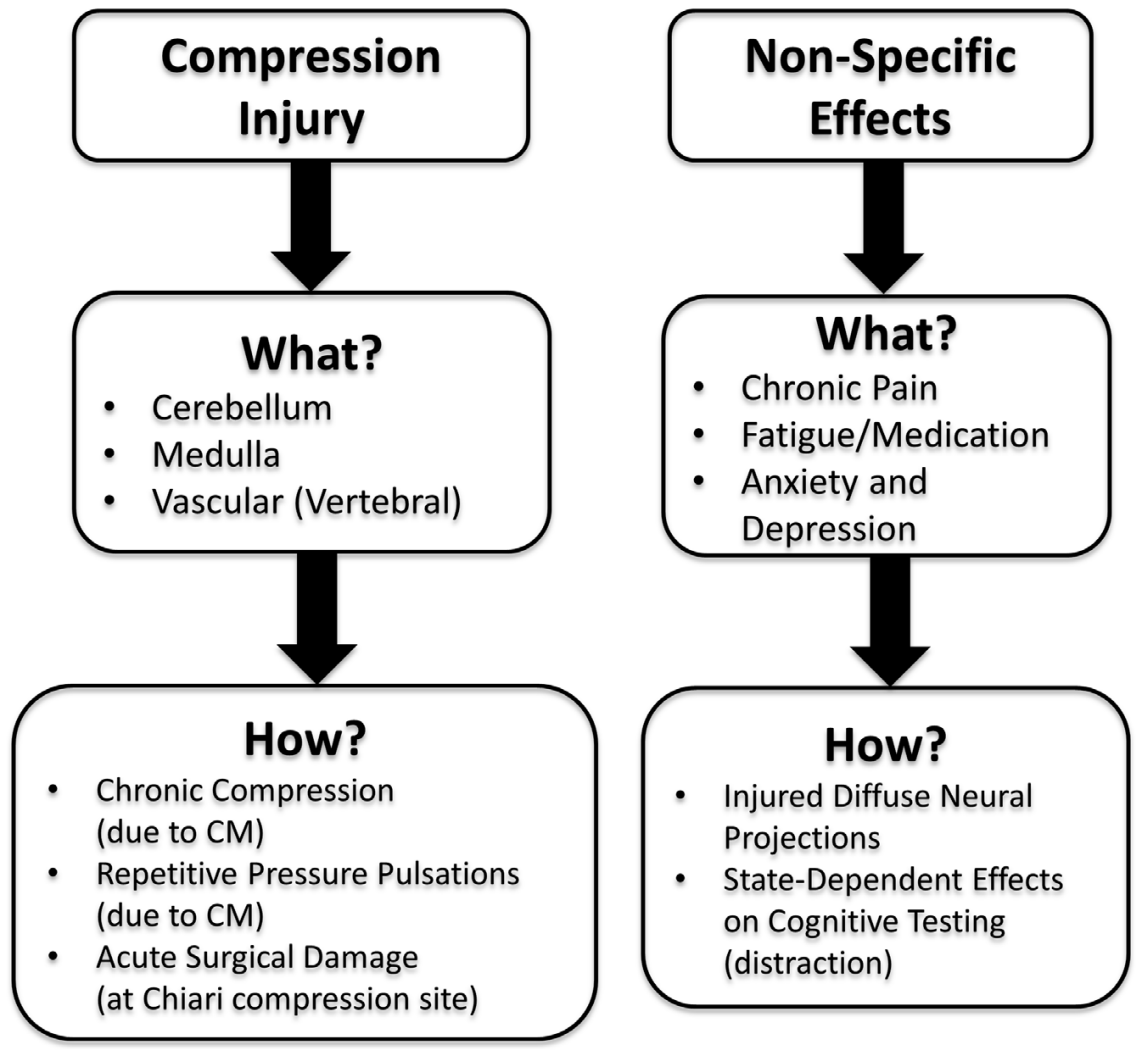
A flow diagram of Chiari I Malformation compression injury and non-specific effects is presented. Under “What,” the anatomical areas or types of non-specific effects are presented. Under “How,” the type of injury or state-dependent effect is presented.

As illustrated in Figure 6, there are several possible causes of the presently observed cognitive dysfunction in the CM group relative to the controls. The two broad categories are: compression injury (e.g., chronic compression from CM or acute decompression from surgery-based injury) and non-specific (e.g., chronic pain). Also, the cognitive deficits observed for the CM group in the present study may have been the result of cerebellar tonsillar injury. However, most of the observable cerebellar damage in CM is done to the floculonodular lobe of the cerebellum (i.e., the caudal portion), and altered CSF pressure (cardiac-induced and/or through coughing or Valsalva maneuvers) may also damage other portions of the cerebellum that have known connections with the prefrontal cortex [7]–[12], however this is speculative. Another likely possibility of fiber-tract damage affecting prefrontal cortex (the area associated with executive function) is the medulla. As illustrated in Figure 1 (in the CM MRI), the cerebellum descends and impacts the brainstem (i.e., the medulla is compressed) in CM. Thus, it could be that brainstem damage rather than cerebellar damage is the culprit of potential fiber tract damage to other portions of the brain—such as the prefrontal cortex—resulting in executive dysfunction, or more diffuse cognitive deficits that indirectly affect executive function. There is evidence of fiber-tract connections between the brainstem and prefrontal cortex [13]. Also, it is known that there are medullary projections to the reticular activating system, the limbic system, and ultimately to the prefrontal cortex [41]. Thus, whether it is based on a cerebellar or a brainstem origin (or both), there are known fiber-tract pathways that link these areas to the prefrontal cortex (associated with executive function).

### Limitations

We choose to test decompressed CM patients knowing that this might result in an underestimate of cognitive deficits due to recovery. Alternatively, there may also be a small chance of surgically induced trauma to the already Chiari-compressed area. As a result, some of the deficits seen may have occurred from surgical cerebellar injury rather than CM-based cerebellar compression (see Figure 6). In this event, it remains true that injury to the Chiari cerebellum would be responsible for the higher-level cognitive effects. In addition, a decompressed sample of CM patients was used to lessen pre-operative anxiety effects and to better insure a conclusive CM diagnosis. Previous studies [42] have demonstrated intraoperative sensorimotor (auditory evoked potentials) improvement within CM patients. This observation supports the idea that the use of post-decompression CM patients is a reasonable strategy because improved, rather than poorer information processing resulted from the decompression procedure.

This study employed statistical control to partial out the effects of anxiety and depression rather than using experimental control. While experimental control is always preferred, it is impractical if not impossible for this patient population. In addition, adult-diagnosed CM patients are largely female, but our CM patient group may have a relatively greater number of female participants than male (22 vs. 2) than is typical of adult CM (probably at least 70% female). We did have more males in the control group (9) than in in the CM group (2), but this was because we used a “yoked” control group when possible. That is, we used spouses or other relatives when possible as controls.

### Conclusion

We provided evidence in this study that CM patients showed poorer cognitive performance on reaction time tasks (working memory, inhibitory control, and processing speed) compared to age- and education-matched controls, but that there were no group differences observed in episodic memory. These results are consistent with both a general cognitive deficit and a specific deficit associated with response (Stroop) inhibition in CM. The locus of the observed response inhibition effect has frequently been associated with prefrontal, executive function [29], [30]. However, the present finding that this response inhibition effect remained statistically significant even after statistical control of anxiety and depression effects, as well as general slowing, whereas other known executive function tasks such as working memory and processing speed were not, provides another potential explanation. For example, it suggests that the observed response inhibition deficit may be more influenced by known areas of damage in CM—namely the brainstem and cerebellum. It is important to note, though, that brain-imaging evidence for localized brain damage in CM for areas other than the cerebellum or brainstem (e.g., the prefrontal cortex) is needed to confirm this speculation. So far, Kumar et al. [3] have provided the only evidence of white-matter integrity losses (based on DTI data) in CM with a relatively small sample size, so more evidence is needed to confirm this possibility.

Finally, while there were task-specific group differences observed for response inhibition, we also observed a multivariate effect of group for all the reaction time tasks and the two episodic memory tasks, and this effect remained significant after statistical control of anxiety and depression. These MANOVA and MANCOVA results suggest that there is also a “global” cognitive deficit in CM.
